# Difficulties Encountered by Forensic Pathologists in Proving Abusive Head Trauma in Children: A Case Report

**DOI:** 10.7759/cureus.49697

**Published:** 2023-11-30

**Authors:** Pavel Timonov, Antoaneta Fasova, Ilina Braynova, Ivan Novakov, Elena Poryazova

**Affiliations:** 1 Forensic Medicine, University Hospital St. George, Plovdiv, BGR; 2 Forensic Medicine and Deontology, Medical University Plovdiv, Plovdiv, BGR; 3 Anatomy, Histology and Embryology, Medical University Plovdiv, Plovdiv, BGR; 4 Forensic Medicine and Deontology, Medical University Sofia, Sofia, BGR; 5 Special Surgery, Medical University Plovdiv, Plovdiv, BGR; 6 General and Clinical Pathology, Medical University Plovdiv, Plovdiv, BGR

**Keywords:** child abuse, diffuse axonal injury, retinal hemorrhage, abusive head trauma, shaken baby syndrome

## Abstract

Shaken baby syndrome (SBS) or abusive head trauma (AHT) is one of the most common causes of death or serious neurological injury resulting from child abuse. AHT is defined as injury to the skull or intracranial contents of an infant or child younger than five years due to intentional abrupt impact and/or violent shaking. It is characterized by acute encephalopathy with subdural and retinal hemorrhages. We present a case of AHT that does not show the typical clinical triad. We describe one case of a pediatric patient addressed for forensic autopsy and where suspicion of AHT has arisen. The injury mechanism involved in the production of this syndrome and its clinical manifestation are sources of debate in forensic medicine. Thus, forensic pathologists must analyze all findings to determine SBS/AHT.

## Introduction

Shaken baby syndrome (SBS) is a serious form of physical child abuse, characterized by acute encephalopathy with subdural and retinal hemorrhages, occurring in the context of inconsistent medical history and commonly accompanied by other inflicted injuries according to Case et al. [1]. The most commonly inflicted injuries, that characterize Child Abuse Syndrome, are fingertip bruising, bite marks, cigarette burns on the skin, spiral fractures of long bones, metaphyseal corner fractures, etc.

This syndrome is detected most commonly in children under two years old. In 1971, neurosurgeon Gutkelch [2] announced, that intracranial and intraocular bleeding in children with no external signs of injury was the result of shaking [2]. Additionally, there is a basic term as “Abusive Head Trauma” (AHT), which represents craniospinal injuries caused by a violent attack on a child, whether by forceful shaking, blunt force trauma, or a combination of both [3]. The clinical triad including subdural hemorrhage, retinal hemorrhage, and encephalopathy is pathognomonic for AHT [4] and it so named by the AAP Committee COCAN (American Academy of Pediatrics and Committee on Child Abuse and Neglect) [4]. Despite these controversies related to used terms Child Abuse Syndrome is a challenge for each forensic pathologist, pediatric neurosurgeon, emergency physician, etc. They must put all clinical and forensic pieces together to prove it.

In the absence of external signs of head trauma, the mechanism of injury is thought to be shaking without a violent blow to the head. When there is evidence of blunt force trauma, it is difficult to determine whether the intracranial injury resulted from blunt trauma alone or the trauma plus shaking. The medical history given by the caretaker is usually vague. Many disorders such as ruptured vascular malformation or cerebral aneurysm [5] or accidental head trauma, which occur after falls [6] can present in a manner similar to AHT. Thus, forensic pathologists and other physicians must be familiar with the clinical and forensic findings of AHT, and its diagnosis is one of exclusion based on close investigation for other cases mimicking child abuse syndrome.

## Case presentation

A one-year and nine-month-old child with loss of consciousness was admitted to the pediatric intensive care unit. His grandmother referred that the child had fallen of the bed. On admission, the child was pale and аtonic and had a Glasgow coma score of 3-4 with fixed mydriasis, corneal and gag reflexes were absent, absence of spontaneous breathing, areflexia, cardiac sinusal rhythm, 130 beats per minute heart rate. Numerous bruises and bite marks were found over different body parts. There wеrе еxternal signs of head injury. The emergency physician described two bruises on the head - left parietal region and right parieto-occipital region. A computerized tomography (CT) head scan showed a global cerebral edema with basal cisterns effacement and a diffuse subarachnoid hemorrhage. MRI examination of brain with different sequences in the evaluation of DAI was not performed on the patient. Ophthalmologic examination discovered bilaterally a lot of preretinal, subretinal and retinal hemorrhages, especially presence of cherry hemorrhages and perimacular ridges. They range from the smallest dot and blot hemorrhage to massive subhyaloid hemorrhage. A skeletal survey ruled out the presence of fractures. The consultation sheet showed that the child was hospitalized for 16 days and during his hospital stay he was intubated and mechanical ventilated. During the hospitalization from the medical report it turned out that the evolution was unfavorable with the installation of an irreversible cardiorespiratory arrest at the resuscitation maneuvers and the child was declared dead.

Macroscopic and microscopic examinations

Eye Injuries

The external examination of the eyes showed that they were intact. We used the posterior approach via the cranial cavity to remove the eye and the contents of the orbit [7]. Their preparation for histological examination related to used technical methods is described in detailed published texts [8]. The representative sections were stained with hematoxylin-eosin.

**Figure 1:**
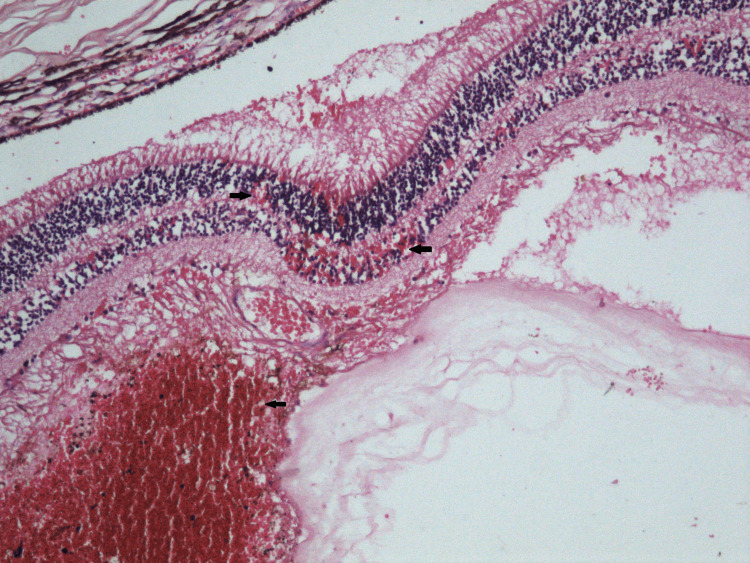
Retinal hemorrhages in inner nuclear layer, a few erythrocytes are seen in outer nuclear layer. Hemorrhage is seen also in internal limiting membrane (H-E, ×100).

The ocular histopathologic observations from this case proved the presence of preretinal, subretinal and retinal hemorrhages. The intraretinal hemorrhages were found within the outer nuclear and outer plexiform layers of the retina. The cherry hemorrhages and perimacular ridges were detected together with a hemorrhage into the internal limiting membrane (Figure 1). Our ocular findings were bilaterally located. The optic nerve sheaths were intact. Retinal detachments were not detected.

Head Injuries

Galea, periosteum, and bone were examined for evidence of injury. We detected two bruises on galea involving left parietal region and right parieto-occipital region. Тhe microscopical characteristics of hairs overlying damaged head areas were examined by a high-quality transmitted light microscope in the range of approximately 40x to 400x. Head hairs were intact without any deformations. 

Skull fractures were not detected. The brain was examined after putting it in 10% formalin for three weeks to better identify the traumatic brain injury [9]. Macroscopic intradural hemorrhages were visible. They were extensive in the falx and tentorium. Diffuse subarachnoid hemorrhages over the right and left cerebral convexities were found. No subdural hemorrhage was identified. Diffuse edema was manifested by flattened gyri and narrowed sulci. Macroscopic examination revealed multiple brainstem pinpoint hemorrhages which were prevalent in its dorsolateral quadrant. 

**Figure 2:**
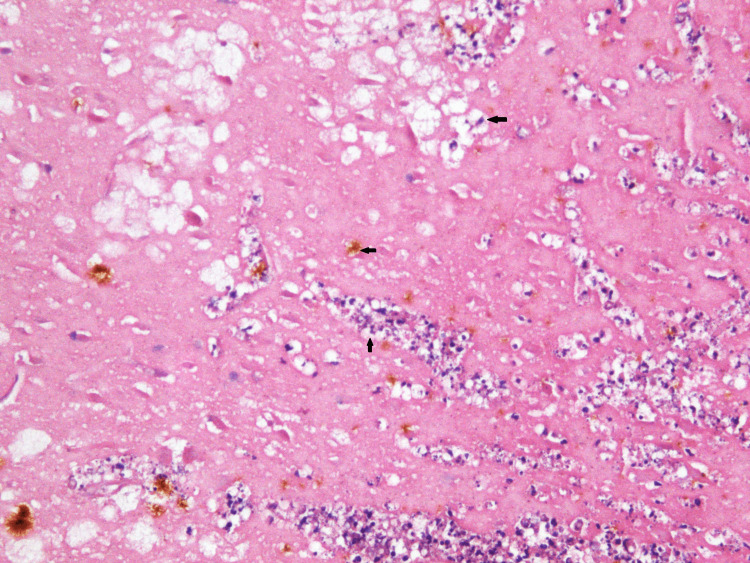
Severe diffuse axonal injury with macrophage proliferation and microglial involvement, single hemosiderophages and foamy macrophages (H-E, ×100).

Sampled regions include the brain parts based on protocol for microscopic brain injury in trauma [10] - anterior corpus callosum, splenium of corpus callosum, posterior limb of internal capsule, hippocampus, temporal, parietal, occipital and calcarine cortex and midbrain. All tissue sections were stained with hematoxylin and eosin. Microscopically, a diffused pericellular and perivascular edema with surrounding microglial cells and macrophages was seen. Multifocal microscopic frontal and temporal white matter lesions were characterized by axonal necrosis (axon loss associated with foamy macrophages) with macrophage proliferation and single hemosiderophages (Figure 2).

The anatomo-pathological macroscopic and microscopic examination of structures of the neck established absence of typical findings for whiplash injury. Findings of multiple durations of injuries are pathognomonic of child abuse syndrome. In this case, they were not presented.

## Discussion

According to Bhardwaj et al. [11], the AHT is defined by subdural hematoma, brain edema, and retinal hemorrhage and is the main cause of subdural hematomas in younger children [11]. This injury is the red flag that is found in victims and is described in 83% to 90% of cases by Feldman et al. [12]. It has been suggested by Geddes et al. [13] and Scheimberg et al. [14] that intradural hemorrhage are common in the perinatal, neonatal, infant, and early childhood age groups, including in alleged cases of AHT [13,14]. It is most commonly found in falx cerebri and tentorium cerebelly according to Cohen and Scheimberg [15] as in the case mentioned above. The brain swelling and raised central venous pressure may cause blood leakage from the venous plexus into the dura. Diffuse cerebral axonal injury (AI) was established by the presence of multiple white matter lesions with a characteristic distribution. Oehmichen et al. [16] stated that DAI was not found in both SBS and control cases and localized AI was regularly present in the brain of the SBS infants surviving more than 1.5-3.0 hours, but only occasionally in the craniocervical junction and within the nerve roots of the upper cervical cord. It was never found in the medulla oblongata [16]. In his study, Plunkett [17] stated that axonal damage is unlikely to be the mechanism for lethal injury in a low-velocity impact such as from a fall [17]. In addition, the presence of external signs of head trauma proves AHT.

According to Green et al. [18] examination of the eyes and optic nerves is crucial in forensic investigations, as in the case of AHT, to demonstrate the specific distribution of hemorrhages and focal areas of the detachment of the retina [18]. The different ocular findings in AHT were analyzed by Riffenburgh [19] who reviewed over 400 autopsy cases of known and suspected child abuse in the Los Angeles region [19]. The author found an absence of retinal hemorrhages in more than 50% of cases, and there were several cases of unilateral retinal hemorrhages. He reported many cases of hemorrhages in optic nerve sheath but without retinal hemorrhages and retinal hemorrhages without optic nerve sheath hemorrhages [19]. Our ocular histopathologic examination proved the presence of bilateral preretinal, subretinal, and retinal hemorrhages without optic nerve sheath hemorrhages. Yamazaki et al. [20] investigated several modes of violent shaking using a dummy doll with an eyeball model which reproduces abusive events that lead to retinal hemorrhages like in SBS or AHT. The authors stated that the time integral of the stress through a single cycle of shaking was 107 Pa·s, much larger than that of a single event of fall, which resulted in 60-73 Pa·s [20]. The abusive shaking is likely to include multiple cycles, and the time integral of the stress due to abusive shaking can be even larger [20]. Based on this statement Yamazaki et al. explained why retinal hemorrhages in SBS/AHT are frequent, while RH in accidental falls is rare [20]. On the other hand, Plunkett [17] announced that retinal hemorrhage may occur when intracranial pressure exceeds venous pressure or when there is venous obstruction. Thus, the characteristics of the bleeding cannot be used to determine the ultimate cause [17].

## Conclusions

Pathologists must pay attention to the anatomic and developmental differences between the brain and skull of children that make the head injuries and mechanisms of injury different in certain respects from those occurring in adults. They are the leading cause of death and disability in children.

There is no specific sign or clinical triad that can establish SBS/AHT. Forensic pathologists and other physicians must analyze all findings. This case shows varied clinical manifestations of SBS/AHT - brain edema, DAI, intradural hemorrhage, diffuse subarachnoid hemorrhage, and bilateral retinal hemorrhages. This is why Child Abuse Syndrome is a serious social and medical problem that many doctors from different medical specialties could face in their routine practice. 
